# Interaction between α-Synuclein and Bioactive Lipids: Neurodegeneration, Disease Biomarkers and Emerging Therapies

**DOI:** 10.3390/metabo14070352

**Published:** 2024-06-22

**Authors:** Chiara Sanluca, Paolo Spagnolo, Romina Mancinelli, Maria Ilenia De Bartolo, Marina Fava, Mauro Maccarrone, Simone Carotti, Eugenio Gaudio, Alessandro Leuti, Giorgio Vivacqua

**Affiliations:** 1 Department of Medicine, Laboratory of Microscopic and Ultrastructural Anatomy, Campus Bio-Medico University of Rome, Via Alvaro del Portillo 21, 00128 Rome, Italys.carotti@policlinicocampus.it (S.C.); 2 Biochemistry and Molecular Biology Unit, Department of Medicine, Campus Bio-Medico University of Rome, Via Alvaro del Portillo 21, 00128 Rome, Italy; 3 Department of Anatomic, Histologic, Forensic and Locomotor Apparatus Sciences, Sapienza University of Roma, 00185 Rome, Italyeugenio.gaudio@uniroma1.it (E.G.); 4 IRCCS Neuromed, Via Atinense 18, 86077 Isernia, Italy; mariailenia.debartolo@uniroma1.it; 5 European Center for Brain Research/IRCCS Santa Lucia Foundation, Via del Fosso di Fiorano 64, 00143 Rome, Italy; mauro.maccarrone@univaq.it; 6 Department of Biotechnological and Applied Clinical Sciences, University of L’Aquila, 67100 L’Aquila, Italy

**Keywords:** α-Synuclein, synucleinopathy, neuroinflammation, neurodegeneration, bioactive lipids, gut-to-brain axis

## Abstract

The present review provides a comprehensive examination of the intricate dynamics between α-synuclein, a protein crucially involved in the pathogenesis of several neurodegenerative diseases, including Parkinson’s disease and multiple system atrophy, and endogenously-produced bioactive lipids, which play a pivotal role in neuroinflammation and neurodegeneration. The interaction of α-synuclein with bioactive lipids is emerging as a critical factor in the development and progression of neurodegenerative and neuroinflammatory diseases, offering new insights into disease mechanisms and novel perspectives in the identification of potential biomarkers and therapeutic targets. We delve into the molecular pathways through which α-synuclein interacts with biological membranes and bioactive lipids, influencing the aggregation of α-synuclein and triggering neuroinflammatory responses, highlighting the potential of bioactive lipids as biomarkers for early disease detection and progression monitoring. Moreover, we explore innovative therapeutic strategies aimed at modulating the interaction between α-synuclein and bioactive lipids, including the development of small molecules and nutritional interventions. Finally, the review addresses the significance of the gut-to-brain axis in mediating the effects of bioactive lipids on α-synuclein pathology and discusses the role of altered gut lipid metabolism and microbiota composition in neuroinflammation and neurodegeneration. The present review aims to underscore the potential of targeting α-synuclein-lipid interactions as a multifaceted approach for the detection and treatment of neurodegenerative and neuroinflammatory diseases.

## 1. Introduction

The interactions between alpha-synuclein (α-Syn) and lipids represent a fascinating frontier in the study of cellular physiology and pathology, particularly in the context of neurodegenerative diseases known as synucleinopathies, which are characterised by α-Syn misfolding and aggregation in neuronal and glial cells. In this review article, we delve into the intricate relationship between α-Syn, a small presynaptic neuronal protein, and various lipid species, elucidating their critical roles in both healthy cellular function and the development of pathological states. Moreover, we aim to explore the multifaceted interactions between α-Syn and lipids across the different pathways involved in the gut-to-brain axis, also defining the potential of α-Syn–lipid interactions in the discovery of novel biomarkers and potential therapeutic strategies for synucleinopathies.

α-Syn is primarily located at the presynaptic terminals of neurons, where it plays a crucial role in the regulation of neurotransmitter release, synaptic function, and plasticity. It is intrinsically disordered in nature, meaning that in aqueous solutions it does not adopt a fixed three-dimensional structure. This structural flexibility allows α-Syn to interact with a variety of biological molecules, most notably with lipids. The interaction between alpha-synuclein and lipids is not only fundamental to its physiological function but also to the pathological aggregation that characterises synucleinopathies. These interactions are critical for the maintenance of synaptic vesicle pools and for the regulation of vesicle trafficking and neurotransmitter release. In addition, α-Syn–lipid interactions are essential for the maintenance of mitochondrial function and integrity, where it has been shown that α-Syn can bind to mitochondrial membranes, affecting mitochondrial dynamics. The pathological significance of α-Syn–lipid interactions emerges when these normally functional engagements become dysregulated. Misfolded and aggregated forms of alpha-synuclein, which are hallmarks of Parkinson’s Disease (PD) and other synucleinopathies, have been shown to have altered interactions with lipids. These pathological interactions can disrupt membrane integrity, impair vesicle trafficking, and induce mitochondrial dysfunction, contributing to cell death. Interestingly, the propensity of α-Syn to form aggregates is influenced by its lipid environment, since certain lipid compositions can promote its pathological aggregation, while others may inhibit it. Emerging research has also highlighted the role of lipids in the seeding and spreading of α-Syn pathology. Lipid rafts, which are microdomains within cellular membranes rich in cholesterol and sphingolipids, have been implicated in the process of α-Syn aggregation and cell-to-cell transmission of pathological species. A process that is central to the progression of α-Syn-related neurodegeneration. Moreover, the study of the lipid composition and metabolism in the gut-to-brain axis and liver is critical in elucidating the mechanisms through which α-Syn spreads from the periphery to the brain, as well as to design specific biomarkers targeting different subtypes of Parkinson’s disease (PD).

The interaction between α-Syn and lipids is crucially involved in both the physiological functioning of neurons and in the development of synucleinopathies, and understanding the molecular bases of this intricate relationship is of the utmost importance to both characterize the pathways that initiate neurodegeneration and for the discovery of potential disease biomarkers or therapeutic strategies in diseases that are caused by aberrant aggregation of α-Syn.

## 2. Genetics and Biochemistry of Synucleins at the Base of Their Preferential Interaction with Lipids in Physiological and Pathological Conditions

α-Syn is a 140 amino acid protein that represents a prominent member of the synuclein family, which also includes β and γ synucleins (β-Syn and γ-Syn) [1]. The three synucleins were discovered and isolated separately from different vertebrate species, with the first dating back to 1988, by Maroteaux and coworkers [2], who isolated a new protein from the electric organ of *Torpedo californica* and found a similar gene sequence in a rat DNA library. These authors called the new molecule “synuclein” due to its prevalent localization at the pre-synaptic (syn) and nuclear (nuclein) levels. A few years later, Nakajo and collaborators isolated and described a specific protein of the nervous system in the brain of *Bos taurus* [3] and gave it the name PNP-14 (phospho-neuro-protein 14) for its high content in phosphorylated residues. On the other hand, the first evidence of α-syn in the human central nervous system (CNS) occurred in 1993, with the isolation of a protein included in the amyloid plaques of patients affected by Alzheimer’s disease (AD), and was therefore defined as NACP (non-amyloid-β component protein) [4]. Finally, in 1995, George and collaborators [5] described a protein in the nervous system of *Serinus canaria*, mainly localized at the pre-synaptic level and up-regulated in specific brain areas during the song-learning period. The authors named this protein “synelphin” due to its peculiar pre-synaptic localization and found strong sequence similarities with proteins previously isolated in *Torpedo californica* (synuclein) and in humans (NACP).

The current distinction into α- and β-synuclein is due to the work of Jakes and collaborators [6], who identified two proteins similar to synucleins in CNS human samples. One of them closely resembled the form described by Maroteaux in 1988—which corresponded to the NACP component of AD—while the other one displayed striking similarities with the PNP-14 protein, previously isolated by Nakajo and collaborators back in 1990 [3]. For this reason, the two proteins were called α- and β-syn, respectively. A third variant—structurally homologous to the original form isolated in *Torpedo californica*—was later found in both rats [7] and humans [8], and was added to the family as γ-syn.

α-, β- and γ-syn genes are located at specific loci on different chromosomes. In particular, the SNCA gene (which encodes for α-Syn), is located at the q21.3–q22 region of chromosome 4 [9,10,11]: mutations of this gene, also called PARK1, are associated with dominantly-inherited PD [12,13,14], while specific point mutations and polymorphisms of its sequence are associated with the pathogenesis of other neurodegenerative diseases such as amyotrophic lateral sclerosis (ALS) and multiple system atrophy (MSA) [15,16,17].

β-syn is encoded by the SNCB gene, which is located on chromosome 5 in the q35 region [10,18,19,20], while γ-synuclein is encoded by the SNCG gene, mapped on chromosome 10 in the q23 region [21]. To date, no evidences have been described linking a mutation of neither of these two genes with any specific human pathology, the only exception being represented by an increase of SNCG gene expression in poor-prognosis breast cancer [8].

**Figure 1:**
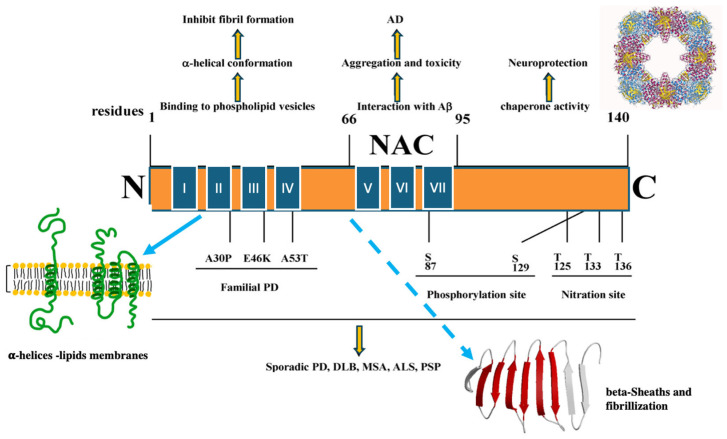
Molecular structure and functional characteristic of human α-Syn (human SNCA gene). α-Syn is functionally divided into N-terminal (1–65aa), NAC (66–95aa), and C-terminal (96–140aa) domains. The N-terminal domain contains four of the seven KTKEGV motifs (dark blue color) and has three-point mutation sites linked to autosomal dominant early-onset PD. It has a prominent propensity to form alpha-helices and bind to lipid membranes. The NAC domain, which encompasses the most hydrophobic residues, has additional KTKEGV motifs, promotes α-Syn aggregation, with a phosphorylation site and the propensity to form beta-sheath conformers. The C-terminal domain exhibits chaperone activity that tends to decrease protein aggregation and has one phosphorylation site and three nitration sites.

The product of the SNCA gene in mammals ranges between 134 and 140 amino acids, depending on the species. It is characterised by a high lysine/arginine ratio and is completely free of cysteine and tryptophan. It consists of: (i) a hypervariable C-terminal region, which is rich in acidic residues; (ii) a central region also called “non-amyloid-β component” (NAC), containing the *GAV motif*, which has been involved in aggregation and fibrillation; (iii) an N-terminal region of 65 amino acids. The protein is also characterized by the presence of a recurring 11 amino acid sequence containing the repeat motif: KTKEGV [2,4,22,23], which is repeated a total of seven times (four times in the N-terminal region, and three times in the NAC region). The three-point mutations most frequently associated with genetic Parkinsonism (A30P, E46K and A53T) are located in the N-terminal region of α-Syn, indicating that the altered succession of repeated sequences is crucial not only for α-Syn physiology but also for its involvement in pathology. The structural characteristics described are schematized in Figure 1 [24].

At the structural level, the N-terminal region of α-Syn (amino acids 1–65) has strong sequence similarities with A2-type lipoproteins and with some proteins that plants accumulate during the seed production and maturation. All these proteins typically bind lipid and tend to fold into α-helix-rich domains [25,26]. The presence of repeated motifs (such as the sequence of 11 amino acids described for α-Syn) represents a further structural advantage in assuming this secondary conformation, in that high amounts of α-helices in the secondary structure is fundamental for the binding of α-Syn to synaptic vesicles: indeed, phospholipid-induced α-helicity increases from 3% to 70% upon binding vesicles, whereas reduced α-helix content at the hydrophobic face of the protein, results in the inability to bind to vesicles, featuring certain phospholipid signatures (e.g., those rich in phosphatidylserine [27]). On the other hand, aggregates of α-Syn shift towards beta-sheath-rich conformations, which prevents their efficient binding to lipid vesicles and leads to the formation of amyloid fibrils that are also responsible for releasing seeding-competent species [28].

Furthermore, unlike SNCB and SNCG, the α-Syn gene SNCA is expressed in different splicing variants, which result in many alternative isoforms of the protein. The 5′ region of the SNCA gene contains an exon with two alternative splicing sites, which can give rise to two possible different variants (Exon 1 and Exon 2). Similarly, alternative splicing has been reported for exons 4 and 6 of the protein [4,9]. These different splicing variants might have different physiological roles and could engage different proteins or lipids, modulating their function.

The central—NAC—region (amino acids 66–95) is specific to α-Syn, it has a fundamental hydrophobic behavior, and is related to the ability of α-syn to create amyloid fibrils, playing a central role in its its neuropathological properties. In fact, the NAC region is not present in β-synuclein, explaining its poor attitude to form insoluble aggregates [6]. Furthermore, α-Syn features seven amino acid motifs of 11 residues (XKTKEGVXXXX), which constitute the structural backbone of the α-helices, involved in vesicle binding (Figure 1): four of these repeats are found in the amphipatic N-terminal region of the protein, while the remaining three are located in the hydrophobic NAC region, with repeats VI and VII being fundamental for the secondary folding of α-Syn and for fibrillization [29].

The C-terminal region (amino acids 96–140) is rich in acidic residues and consists of repeats of 16 amino acids, which seemingly play an important role in calcium binding [30]. This region is also highly specific to α-Syn, showing consistent differences in the primary sequence compared to the homologous regions of β- and γ-syn [23]. The C-terminal region has anti-fibrillogenic properties, as demonstrated by the fact that the truncated forms of α-Syn, lacking this region, are much more prone to form insoluble fibrils than the “full length” species [31]. Indeed, the C-terminal region would perform an intramolecular chaperone function aimed at guiding the physiological folding of α-Syn and preventing the fibrillogenic misfolding [32,33]. The C-terminal region would also have protective properties against oxidative stress, as demonstrated by the fact that truncated forms, lacking the C-terminal region, correlate with an increased sensitivity to oxidative stress in dopaminergic neurons [34].

α-Syn is subject to numerous post-translational enzymatic modifications, including phosphorylations or nitrations. These modifications occur at specific sites of the molecule (Figure 1) and contribute to both the physiological and pathological activity of the protein. In particular, the phosphorylation of Ser129 or the nitration of Tyr125, Tyr133 and Tyr136 correlate with an increased tendency of α-Syn to aggregate into fibrils [35,36,37].

Fibrillar and filamentous α-Syn with a beta-sheet secondary conformation is the main constituent of Lewy bodies and Lewy neurites [38,39], where this misfolded protein is associated with a crowd of broken and degenerated lipid membranes [40]. The term “oligomer” is instead widely used to describe aggregated α-Syn that has not necessarily acquired a fibrillar β-sheet conformation; the term itself is rather unspecific with the molecular composition of α-Syn oligomers, encompassing a wide spectrum of molecular weights, β-sheet content and exposed hydrophobicity. Low molecular weight and unstable oligomers are defined as “off-pathway” oligomers: they only have marginal seeding effects, although they can impact neuron functionality in several ways (e.g., synaptic dysfunction, perturbation of lipids membranes, triggering apoptosis and ER stress), leading to neurodegeneration [41,42]. On the other hand, large and stable oligomers are usually elongated and disclose a typical beta-sheet structure, which defines them as “on-pathway” oligomers with high-seeding properties [41,42,43].

It is important to note that although physiological α-Syn has largely been considered a natively unfolded monomer that acquires an alpha-helical secondary structure when interacting with lipids membranes [44], it was recently suggested that native α-Syn exists physiologically as a folded helical tetramer that is resistant to fibrillization and is thus distinct from pathological oligomers [45,46,47,48]. In this regard, recent data from Burrè and coworkers [49,50] using FRET and cross-linking, demonstrated that an equilibrium exists between monomeric cytoplasmic α-Syn and multimeric membrane-bound α-Syn that acts as a SNARE chaperone. The conformation of physiological α-Syn remains a contentious issue but understanding the identity of the native form, or whether there are multiple native conformers in equilibrium in the cell, is important to inform the development of potential anti-aggregation therapies for PD.

## 3. Alpha-Synuclein and Biological Membranes: The Importance of Protein-Lipids Interactions

α-Syn is primarily located at the presynaptic terminal, where it plays a complex and multifaceted role in vesicle docking and neurotransmitter release during synaptic transmission. One of its primary functions is to regulate the availability of synaptic vesicles, hence playing a critical role in maintaining the pool of readily-releasable vesicles at the synaptic terminal. At this site, it interacts with many lipids and facilitates the assembly of the soluble N-ethylmaleimide-sensitive factor attachment protein receptor (SNARE) complex, which is responsible for the fusion of the synaptic vesicle with the plasma membranes. These interactions potentially result in a conformational change in the local membrane environment, inducing the membrane curvature required for vesicle formation and fusion. In the SNARE complex, α-Syn binds to the protein synaptobrevin-2/vesicle-associated membrane protein 2 (VAMP2), and drives SNARE-mediated vesicle fusion, facilitating vesicular docking to the presynaptic plasma membrane thus driving neurotransmitter release [24,51]. Abnormal and aggregated α-Syn impedes its normal capacity to regulate the SNARE complex, disrupting vesicle docking. This synaptic dysfunction is considered a primary cellular alteration in PD [52].

Several studies have elucidated the mechanism of α-Syn interaction with lipid membranes, including synaptic vesicles, which are naturally occurring structures enclosed by a bilayer of phospholipids responsible for the release and reuptake of neurotransmitters.

The interaction of α-Syn with lipid membranes is initiated when the N-terminal domain of synuclein forms an extended—or broken—alpha-helical structure which depends on the phospholipid-to-protein ratio and membrane curvature. Indeed, the elongated alpha-helical conformation tends to be induced upon interaction with membranes with a lower degree of curvature and a diameter of approximately 100 nanometers; on the other hand, a broken helical conformation is described in the presence of highly curved, small vesicles [53] and is crucial to maintain the correct curvature of the vesicles, necessary for synaptic docking and fusion.

Distinct lipids subtypes differentially interact with α-Syn at the level of lipid membranes [51,54]: indeed, structural studies have demonstrated that the N-terminal domain of the protein is rich with positively charged lysine residues, explaining why its lipids affinity is heavily increased by the presence of lipids with acidic and negatively charged groups. This high propensity of α-Syn to interact with negatively charged lipid membranes has been also studied as a possible therapeutic strategy for synucleinopathies, as in the case of the small molecule Anlel138b [55], where the drug was loaded into negatively charged liposomes, namely POPA (1-palmitoyl-2-oleoyl-sn-glycerol-3-phosphate) and POPC (1-palmitoyl-2-oleoyl-sn-glycerol-3-phosphocholine). These two liposomes contain anionic phospholipids, with a higher tendency to bind α-Syn aggregates, creating a privileged site of action for the drug in preventing synaptic dysfunction and α-Syn spreading [56,57,58].

An important site for the α-Syn–lipid interaction occurs at the level of mitochondrial membranes. Cardiolipin, a phospholipid localized at the inner mitochondrial membrane, plays a key role in mitochondrial function and structure, with its unique structure – characterized by four acyl chains and two phosphate groups—allowing it to interact with proteins that cannot bind to regular phospholipids. Upon interaction with certain lipids, such as cardiolipin, α-Syn can gain α-helical structure [26] and the N-terminal region of α-Syn, incorporating the repeat motif, rich in KTKEGV residues, is believed to mediate the interaction of α-Syn with cardiolipin [54], through the promotion of electrostatic interactions and the formation of additional α-helical domains [59]. Under normal conditions, the interaction between α-Syn and cardiolipin contributes to normal mitochondrial functions, potentially including the regulation of mitochondrial morphology and the process of bioenergetics. Increased penetration of α-Syn into mitochondria, as it occurs in the course of synucleinopathies, can instead destabilize the mitochondrial membrane and compromise its function [60]. Alterations in cardiolipin content or structure can enhance the binding of α-Syn to the mitochondrial membrane, and lead to increased oxidative stress and, eventually, to neuronal cell death [60,61]. Mutations in α-Syn that result in increased binding affinity to cardiolipins, are associated with familial forms of PD [62]. Also of note, the interaction between α-Syn and cardiolipins can lead to the formation of pore-like structures, which might contribute to cytochrome c release and apoptosis of DA neurons, a key feature in PD [63].

Different biochemical factors affect the interaction of α-Syn with lipid membranes and contribute to its physiological and pathological properties. Among them, the biochemical properties of the lipid bilayer, the conformational state of α-Syn and its post-translational modifications, the dynamics of α-Syn–lipids interactions and the genetic mutations of α-Syn are worth discussing.

The biochemical properties of the lipid bilayer significantly affect the interactions with α-Syn. The cell membrane comprises two layers of phospholipids with distinct head groups, embedded cholesterol and floating proteins. The membrane fluidity state depends, among several factors, on the length of the hydrocarbon tails: if these are short, there is a reduced tendency of the hydrocarbon tails to interact with the others, leading to increased membrane fluidity; conversely, if the tails are longer, the chances to interact between them are higher, conferring to the membrane a more compact structure. The fluidity is also influenced by the number of double bonds in the phospholipid-bound fatty acids and by the presence of cholesterol. This constitutes around 20% of the lipid content of the membrane and can fill the spaces between the kinks of the unsaturated hydrocarbon chains, contributing to increasing the membrane rigidity. Some studies report that cholesterol plays a role also in the packing density of lipids, decreasing the likelihood of lipid vesicles being disrupted upon interaction with toxic α-Syn aggregates [64]. Other studies highlighted, instead, that cholesterol can promote the binding of α-Syn oligomers to the membranes, thus increasing α-Syn toxicity [65]. However, these conflicting hypotheses are not necessarily mutually exclusive, since the role of cholesterol in the interaction of α-Syn with lipid membranes is complex and can be beneficial or cytotoxic depending on the phospholipid’s composition of the lipid membranes.

As explained previously, in fact, α-Syn monomers demonstrate a high affinity for negatively charged lipids due to the positive electrostatic interactions between the positive charges on their N-terminal domain and the anionic groups of the lipid bilayer [51,54,66]. In line with this concept, lipids vesicles with a high content of phosphatidylethanolamine or phosphatidic acid disclose a greater propensity to bind α-Syn in comparison to those with a higher expression of phosphatidylserine and phosphatidylcholine which present cationic functional groups [67,68]. As for α-Syn oligomers, they disclose heterogeneity in size, structure and biochemical conformation, which reflects on different interactions with lipid membranes. Notwithstanding, despite their heterogeneity, a common property of α-Syn oligomers is their increased hydrophobicity compared to monomeric α-Syn, which leads to an increased affinity for the hydrophobic tails of phospholipids [41,69]. This property is exacerbated in α-Syn oligomers acquiring a beta-sheath amyloid conformation [70]. Mustaikyte et al. identified two types of α-Syn oligomers (type I and type II) sharing the hydrophobic core region NAC, characterised by a beta-sheath conformation [71]. Moreover, another study carried out by Fusco and coworkers reported the classification of α-Syn oligomers in type A and type B, based on their ability to perturbate the lipid membranes [72]. In particular, type B oligomers, characterised by highly lipophilic beta-sheath elements, are able to perturb biological membranes, disrupting their integrity and forming intra-membrane pores, and are able to interfere with neuronal compartmentalization and synaptic transmission [73,74,75], leading to neurotoxicity. More in detail, several mechanisms have been proposed for the interaction of α-Syn with lipid membranes, including pore-forming interactions, bilayer thinning and detergent-like solubilization [76]. Pore formation highlights how pathological oligomeric α-Syn can influence membrane integrity. α-Syn oligomers can interact with the membrane causing a conformational change that is skewed towards lipid bilayer thinning [75], with a consequent leakage across the membrane. Electrostatic forces between α-Syn and head groups of the lipid bilayer are crucial for this conformational change to occur because they alter the ordered structure of the membrane, reorganizing and leading it to an increased-permeability state. In turn, the altered membrane permeability will manifest as an unbalanced ion leakage resulting in cellular homeostasis disruption with consequent swellings of several organelles. Furthermore, the affinity of synaptic vesicle membranes with α-Syn oligomers is of substantial importance for the transmission and spreading of α-Syn pathology. On-pathway α-Syn oligomers, characterised by beta-sheet structure, have an increased affinity for lipid membranes. Their biochemical conformation confers the seeding capacity and the ability to drive the formation of α-Syn fibrils in contiguous neurons, exploiting the synaptic connections [41,42].

In the setting of protein–lipid interaction, the post-translational modification of α-Syn occupies a relevant role [66]. Not surprisingly, α-Syn undergoes post-translational modifications, which can bring changes in protein hydrophobicity as a consequence of alteration in charge and structure, thus interfering with α-Syn–lipid interaction; prominent examples of such modifications are (i) acetylation, (ii) phosphorylation, (iii) oxidative modifications and (iv) truncations.

Acetylation, occurring both in healthy individuals and in PD patients, consists of the attachment of an acetyl group at the N-terminal domain of the protein, in particular to the alpha-amino group of the first amino acid. Consequently, the typical positive charge of the N-terminal domain is lost, leading to a decreased binding affinity for the membrane anionic lipids [77]. Unlike acetylation, phosphorylation affects primarily disease conditions. Accordantly, 90% of α-Syn fibrils in the context of Lewy bodies are phosphorylated at serine 129 (Ser129), while other residues have been identified as targets of the phosphorylation process. Serine 87 (Ser87) phosphorylation, for example, can be considered a pathological hallmark of α-Syn inclusions. Albeit Ser87 and Ser129 are both substrates for kinase enzymes, their influence on the interaction of α-Syn with lipid membranes differs. Although Ser129 phosphorylation is strongly related to the formation of beta-sheath fibrils, it seems to have only a milder effect on α-Syn–lipid interactions, while Ser87 phosphorylation significantly reduces the binding of α-Syn to lipid membranes [78,79,80,81,82,83]. Interestingly, in vitro studies have also highlighted how the subtype of the enzyme carrying out the phosphorylation can influence the lipids interaction of α-Syn; it has been reported that phosphorylation carried out by G-protein-coupled receptor appears to reduce the membrane binding affinity [77]. The modulation of α-Syn phosphorylation, by acting on G-protein coupled kinases could, therefore, represent a possible therapeutic approach to prevent α-Syn neurotoxicity. Among the oxidative modifications, the most well-studied one is the nitration of α-Syn tyrosine residues [77], which reduces the membrane binding affinity but also appears to play a role in promoting α-Syn oligomerization [66]. Studies also focused on the effect of methionine oxidation on the interplay of α-Syn with lipids in synaptic vesicles; methionine appears to be particularly sensitive to oxidation and methionine oxidation reduces the solvent-induced alpha-helicity of α-Syn, resulting in the stabilization of toxic oligomeric species [84]. Remarkable attention has to be drawn to α-Syn truncation, which more likely occurs on the N-terminal domain of the protein. This region is rich in positive-charged lysine residues, which positively affect the binding to anionic lipid membranes. Therefore, a truncation at this level is expressed with a reduction in α-Syn membrane affinity [85]. Conversely, a less prominent consequence is seen in the setting of C-terminal domain truncation. This domain, in fact, plays a pivotal role in the assembly of the SNARE complex via synaptobrevin-2 biding interaction and it is less involved in the interaction with lipids membranes [85].

The effect of genetic mutation of α-Syn is heterogeneous since not all the mutations have the same impact on the process of α-Syn aggregation and lipids interaction [70]. All mutations of the SNCA gene have been linked to the aggregation of α-Syn, with lipid membrane interaction being influenced at various degrees. In particular, A30P and G51D mutations appear to hinder the interaction of α-Syn with lipid membranes, although their propensity to form α-Syn oligomers [86,87]. Conversely, the A53T mutation was associated with a weak tendency to form oligomers and an increased ability of fibrillization and formation of beta-sheath structures, leading to higher affinity for lipids membranes [88].

## 4. Endogenous Bioactive Lipids and Synucleinopathies

Bioactive lipids represent a rather wide class of endogenous molecules that include four main genres, namely eicosanoids, sphingolipids, endocannabinoids and specialized pro-resolving mediators (SPM). These compounds are generated from—or are related to—the different plasm membrane glycerolphospholipids, from their sn2-bound polyunsaturated fatty acids (PUFA) from other lipids that participate in the overall metabolism of the cell, and are deeply embedded in several physiological or pathological processes, including acute and chronic inflammation, neuroinflammation and its resolution, tumors and autoimmunity, as well as in those molecular pathways that lead to α-Syn aggregation and neurodegeneration [89,90].

**Figure 2:**
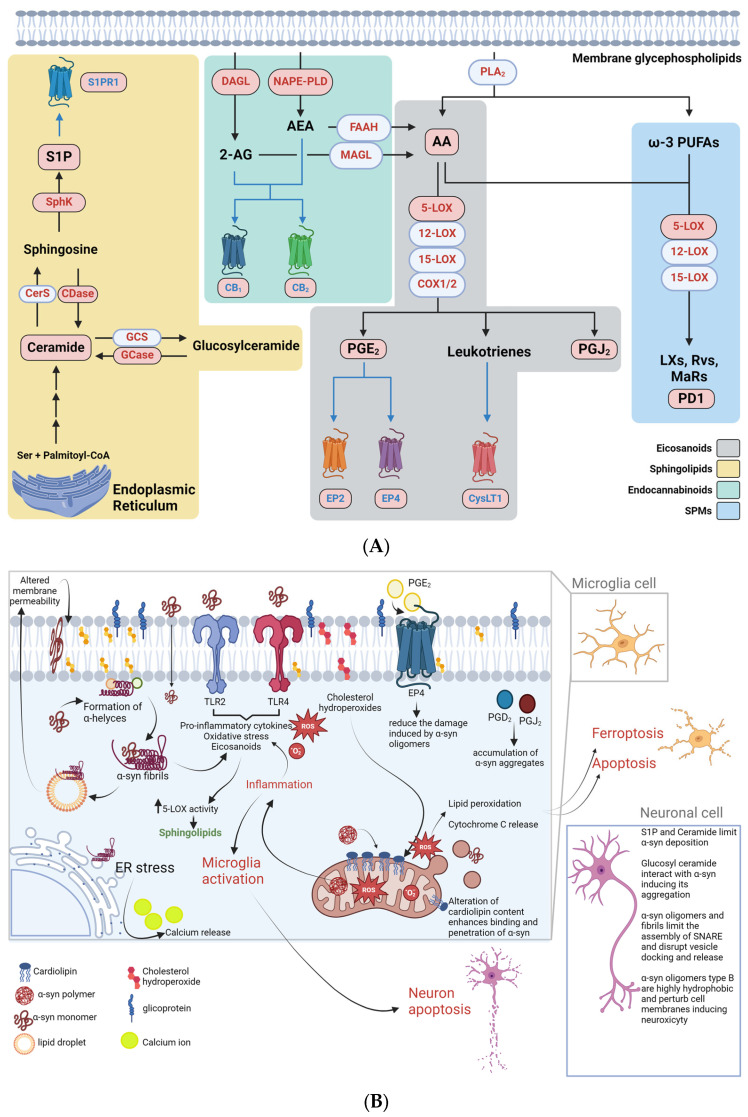
Cellular and molecular pathways involved in α-synucleinopathies. (**A**) Main metabolic pathways of endogenous bioactive lipids. Elements highlighted in pink represent molecular targets that have been involved or investigated in synucleinopathies. (**B**) Pathogenic processes that have been linked to synucleinopathies: Microglia can recognize, uptake and phagocyte α-synucleins. In pathological conditions α-Syn aggregates into oligomers, protofibrils, and fibrils, which further bring to the formation of Lewy Bodies. Misfolded α-Syn has the ability to act as a DAMP and contributes to the build-up of the inflammatory surge by directly activating microglia-mediated responses engaging TLR2 and TLR4, thus leading to the production of pro-inflammatory cytokines, prostaglandins, leukotrienes, and reactive oxygen species (ROS). The pro-inflammatory cytokines will further promote microglial activation leading, in turn, to production of ROS and oxidation of α-Syn in neighboring neurons. Among bioactive lipids, PGE_2_ can also engage anti-inflammatory receptors, such as EP4, which can dampen damage induced by α-Syn oligomers in primary microglial cells. 15-PGDH, 15-prostaglandin dehydrogenase; 2-AG, 2-arachidonoylglycerol; AA, arachidonic acid; AEA, anandamide or N-archidonoylethanolamine; CB cannabinoid receptor; CDase, ceramidase; CerS, ceramide synthase; COX, cyclooxygenase; CysLT1, cysteinyl leukotriene receptor 1; DAGL, diacylglycerol lipase; EP, prostaglandin E_2_ receptor; FAAH, fatty acid amide hydrolase; GCase, glucosylceramidase; GCS, glucosylceramide synthase; LOX, lipoxygenase; LX, lipoxin; MAGL, monoacylglycerol lipase; MaR, maresin; NAPE-PLD, N-acyl phosphatidylethanolamine phospholipase D; PD, protectin; PG, prostaglandin; PLA2, phospholipase A2; PUFA, polyunsaturated fatty acid; Rv, resolvin S1P, sphingosine-1-phosphate; SphK, sphingosine kinase; SPM, specialized pro-resolving mediators.

Of note, the aberrant aggregation of α-Syn has been often linked with altered lipid metabolism, accumulation and homeostasis [51] as well as to the deviant activity of enzymes that can access the lipids of the plasma membrane double layer [91,92]. Of note, the phospholipid composition of the membrane is also related to the physiological role of α-Syn in synaptic function [51,93]. On the other hand, even though bioactive lipids that act as autacoid hormone-like molecules—or the enzymes and receptors that orchestrate their metabolism and signalling—have been reported to participate in proper α-Syn aggregation, they also act by modulating the inflammatory surge that enhances α-syn-driven damage (or that sometimes is kickstarted the of α-Syn oligomers in the first place) [94,95]. It should be noted that this interaction is duplicated, in that α-Syn oligomers can lead to dysfunctional production and metabolism of these endogenous lipids. The metabolic pathways linked to these compounds that are involved in synucleinopathies are summarized in Table 1 and schematized in Figure 2.

### 4.1. Eicosanoids

Eicosanoids are arachidonic acid (AA)-derived lipids that represent the most prominent genus among bioactive lipids, and those that have been studied the most thus far, as the main therapeutic target of all cyclooxygenase 1 and 2 (COX1-2)-inhibiting non-steroidal anti-inflammatory drugs (NSAID) [96]. All eicosanoids are produced when AA that is tethered to membrane glycerophospholipids is released upon the activation of phospholipase A2 (PLA2), before undergoing the COX1/2-dependent oxidation that leads to prostaglandins and thromboxanes or being targeted by lipoxigenases (LOX) in a pathway that leads to leukotriene biosynthesis [97]. As the main effectors in acute and chronic inflammation in both the periphery and in the CNS, eicosanoids have been linked to α-syn-driven neurodegenerative processes: although the molecular mechanisms behind this process have not been completely elucidated yet, it might either happen by the direct action of eicosanoids triggering deviant pathways that promote the accumulation of oligomers, or through the disruption of α-Syn physiological functions, as suggested by the fact that α-Syn ablation leads to decreased brain AA turnover, and increased prostaglandins level after CNS insults such as stroke [98,99]. As a matter of fact, misfolded α-Syn contributes to the build-up of the inflammatory surge by directly activating microglia-mediated responses, possibly by the ability of these cells—and, quite possibly, that of all pattern-recognition receptor (PRR)-expressing antigen presenting cells (APC)—to recognize these aggregates as a pathogen- or damage-associated molecular pattern (DAMP or PAMP, respectively), as demonstrated by the fact that α-Syn can engage TLR2 [100,101] and TLR4 [102], as well as the evidence that the ablation of CD36 from mice-derived glia leads to a less severe activation in these cells [103]. The activation of PRRs in innate cells is strongly linked to the production of reactive species and of pro-inflammatory cytokines/chemokines and lipids—first and foremost eicosanoids. A number of works dating back to the first decade of the 2000s described an important role for prostaglandin E_2_ (PGE_2_) and its signalling in the relationship between eicosanoids and synucleinopathies, the earliest of which reported enhanced prostaglandin (PG) E_2_-mediated neurotoxicity in α-syn-overexpressing neuronal cell lines, as compared to control cells [104], and the enhanced ability of EP2^−/−^ microglia to both clear out α-Syn aggregates and blunt the effect of neurotoxic Parkinsonian drugs used in mouse models [105]. Interestingly, a recent paper explored the predictive value of COX2, EP2 (a PGE_2_ receptor) and α-Syn as biomarkers in the early diagnosis and intervention of autism spectrum disorders [106] reinforcing the link between α-Syn function and that of AA-derived autacoids. On the other hand, PGE_2_ can also engage anti-inflammatory receptors, such as EP4, which has been reported to act in a beneficial way on α-syn-mediated damage: indeed, EP4 agonism is able to dampen the damage induced by α-Syn oligomers in primary microglial cells [107]. Other eicosanoids have also been linked to the molecular mechanisms that lead to the accumulation of α-Syn aggregates: PGD_2_ is a major prostaglandin in the brain, which can undergo non-enzymatic dehydration and yield PGJ_2_, a potent neurotoxic compound [108]. This eicosanoid is not only able to exert a strong glial activation when infused in the brain of animal models, but it also directly leads to the accumulation of α-Syn in rodent brains and in SK-N-SH cells [109]; in particular, in the latter model, the aberrant deposition of ubiquitinated proteins (including α-syn) following PGJ_2_ treatment is also accompanied by a collapse of the cytoskeleton and of the Golgi and ER network, which possibly leads to the relocation of resident proteins into the aggregates [109].

Of note, a more recent cohort of papers has investigated the role of other eicosanoids, such as LOX-derived leukotrienes, in synucleinopathies. These mediators, especially their cysteinylated derivatives, are better known as the endogenous lipids involved in the pathogenesis of asthma, and the target of widely used drugs such as Montelukast, Pranlukast and Zafirlukast, which act as antagonists to CysLT1, the receptor engaging the cysteinylated derivative of leukotriene (LT) B_4_ [110]. However, these eicosanoids have also been recently involved in brain pathologies, including those deriving from misfolded α-syn; indeed, not only leukotrienes are strong activators of astrocytes and microglial cells (as reviewed in [111]), but an increase in the activity of 5-LOX, i.e., the enzyme catalyzing the rate-limiting step in leukotrienes synthesis, has also been found in patients and animal models of DLB [112]. Thus, recent papers have investigated the effect of the Montelukast in DLB, showing beneficial effects on the clinical phenotype and a reduction in the alphα-Syn load in the brains of DLB animal models [112]. Interestingly, Montelukast is also under investigation in a clinical trial (EudraCT: 2020-000148 [113]) as a potential treatment for PD, suggesting that LOX-dependent pathways of AA might represent a promising therapeutic target in the treatment of these diseases.

### 4.2. Sphingolipids

Sphingosine, the prototype and backbone of all sphingolipids, was the first compound of this class to ever be identified, and was named after the Greek mythical creature—the Sphynx—due to the “the many enigmas which it presented to the inquirer” (as reported by its discoverer J.L.W. Thudichum in 1884) [114]. Besides sphingosine, this major lipid class features other compounds like ceramide, the phosphorylated derivatives ceramide-1-phosphate (C1P) and sphingosine-1-phosphate (S1P), as well as other complex sphingolipids such as sphingomyelins and glycosphingolipids. Taken together, these molecules play a pivotal part in brain tissue and immune homeostasis.

Sphingolipids, especially glucosylceramides and glycosphingolipids, are crucial in membrane dynamics and in the organization of cholesterol-rich lipid rafts and have represented a main target of investigation in the α-syn-related pathologies in the last decade. In particular, mutations of the GBA gene, i.e., the gene encoding for the lysosomal enzyme glucosylceramidase (GCase), which hydrolyzes glucosylceramide (also known as glucocerebroside) into ceramide and glucose [115], causes Gaucher disease and represents a major genetic risk factor for PD [116,117]. A number of mechanisms have been proposed that link GBA mutations to the accumulation of α-Syn in PD, most of them converge towards an engulfment or failure of the autophagic or endolysosomal processes that are pivotal in the clearance of the pathogenic aggregates [118]. Indeed mutated—and often misfolded—GCase can lead to its impaired trafficking between the ER, the rest of the cell compartments, or the proteasome, it can exhibit deficient activity that causes glucocerebroside accumulation (as reviewed in [115]). On one hand, glucosylceramide is thought to interact with α-Syn and cause its aggregation—a mechanism that might be exacerbated upon loss of GCase activity and accumulation of its substrate. This hypothesis seems to be reinforced by the evidence that not only early phases of intermittent PD display reduced brain GCase activity, but also that this is majorly evident in areas with higher α-Syn deposition [115]. On the other hand, GCase and α-Syn can reciprocally interact, affecting each other’s molecular properties: indeed α-Syn displaces GCase from the plasma membrane, possibly reducing its access to the substrate, while GCase acts on α-Syn by shifting the residues of a helical domain, which might contribute to its oligomerization and aggregation [91,92]. This mechanism might be exacerbated in case of overexpression of the GBA gene.

Other authors have also recently sought to characterise the involvement of the S1P/Ceramide rheostat in synucleinopathies. In general, S1P and Ceramide are thought to regulate the production and aggregation of proteins involved in neurodegeneration, including α-syn, as also demonstrated by the fact that sphingolipid metabolic disturbances and higher levels of ceramides and sphingomyelins have been found in the brain and plasma of PD patients [119]. Inhibition of sphingosine kinase 1 (SphK1), i.e., the enzyme that catalyzes S1P biosynthesis by sphingosine phosphorylation leads to increased α-Syn secretion and apoptosis in human dopaminergic neurons [120], while pharmacological murine models of PD display reduced activity of this enzyme [121]. The importance of S1P—and its receptor S1PR1—in the pathogenesis of PD might be also inferred by the fact that a few studies that reported a beneficial effect of fingolimod (or FTY720)—an S1PR1 antagonist/modulator and the first oral treatment ever patented in the therapy for relapsing-remitting multiple sclerosis—ameliorates the clinical phenotype in murine models of PD [122].

On the other hand, alterations of ceramide metabolism have been involved in the development of protein aggregates, such as those that contribute to Levy bodies, as demonstrated by the fact that the inhibition of acid ceramidase—which promotes the hydrolysis of ceramide to sphingosine and fatty acids—has been associated with reduced accumulation of α-Syn [123]. Interestingly, polymorphisms of the acidic ceramidase gene (i.e., ASAH1) are associated with PD pathogenesis [124].

Other genes involved in sphingolipid metabolism that have been linked to the pathogenesis of synucleinopathies include SMPD1 (acid sphingomyelinase, i.e., the enzyme converting sphingomyelins into ceramide), and PSAP, which encodes the precursor of the saposin protein that acts as an activator of ceramidases that control the metabolism of complex sphingolipids [124,125].

### 4.3. Endocannabinoids

Endocannabinoids are a group of lipid mediators that act as endogenous ligands to those receptors that are engaged by the psychoactive constituents of *Cannabis sativa* and *Cannabis indica* and have been both characterised as important neuromodulatory and immunomodulatory agents [126]. The so-called “endocannabinoid (eCB) system” includes the two major eCBs, i.e., N-arachidonoylethanolamine (AEA, also known as anandamide) and 2-arachidonoylglycerol (2-AG), their two main receptors, i.e., CB_1_ and CB_2_, as well as the enzymes that control biosynthesis (i.e., *N*-acylphosphatidylethanolamine-hydrolyzing phospholipase D [NAPE-PLD], and *sn*-1-DAG lipase (DAGL)) and degradation (i.e., fatty acid amide hydrolase (FAAH) and monoacylglycerol lipase (MAGL)) [90,126].

It should be noted that in recent years, a number of other receptors have been identified that bind eCBs, such as the nuclear peroxisome proliferator-activated receptors (PPAR) α and γ, the transient receptor potential vanilloid type-1 (TRPV1) ion channel, and GPR55 and GPR119 [39,40], as well as other enzymes that participate in their metabolism, including e αβ-hydrolase domain (ABHD) 2, 4, 6 and 12, as well as COX and LOX enzymes [90].

Further details of eCB metabolism and signalling have been recently reviewed in [90].

To date, we possess scarce data—if any—regarding a direct role of the eCB system in the pathologic aggregation of α-syn, although several works have been published in the past two decades that described an involvement of these lipids in PD, in both mechanistic and therapeutic approaches.

CB_1_ is a widely expressed G protein-coupled receptor that exerts crucial neuromodulatory roles in synaptic control, and there is evidence supporting its involvement in reducing PD bradykinesia, as well as in enhancing the therapeutic effects of levodopa administration [127,128,129]. However, the pharmacological exploitation of this receptor is difficult due to the relevant psychiatric side effects of this approach [130]. CB_2_, on the other hand, seems to represent a rather more promising target in neuroinflammatory contexts, including those involved in PD [131]. Indeed, CB_2_ agonism reduces the loss of TH neurons in SNpc of LPS- and MPTP-based rodent models of PD [132,133], and it is upregulated in response to neuronal damage in this area [134]. Interestingly, the upregulation of CB_2_ in SN is also accompanied by elevated levels of AEA and 2-AG, further suggesting a role for the eCB system in PD [134]. It should be noted, however, that even though these PD models can theoretically feature oligomer accumulation [135,136], the studies reporting α-Syn aggregates were achieved through variant setups involving the chronic administration of neurotoxic agents that cause neuronal loss; on the other hand, the studies addressing the role of the eCB system in PD models were conducted with acute administrations, and none of them observed directly α-Syn aggregates.

The activation of the eCB system can also be achieved in a more “physiological” way, by targeting AEA and 2-AG breakdown enzymes (FAAH and MAGL, respectively). This approach has shown promising results in other neurodegenerative conditions such as Alzheimer’s disease [137,138], but the effect of this strategy on synucleinopathies is still quite scarce and, in some cases, contradicting. In recent work, authors reported that specific inhibition of MAGL, but not FAAH, protects from striatal DA depletion in an MPTP/probenecid-based mouse model of PD [139], possibly suggesting a role for 2-AG in PD. Interestingly, this eCB binds with high-affinity CB_2_. By contrast, a number of other works documented beneficial effects of FAAH inhibition in similar rodent models [140,141,142,143], showing reduced neuronal cell death in basal ganglia or ameliorated clinical phenotypes, raising the possibility that AEA might have a bigger importance, instead. Then again, none of these works addressed the effect of the treatment on the deposition or secretion of α-syn.

Further investigations will be necessary in the near future to better understand the role of eCBs in the pathological accumulation of protein aggregate-based diseases.

### 4.4. Specialized Pro-Resolving Mediators

SPMs are a recently described class of lipid modulators that drive the “resolution of inflammation”, which is the active termination of acute inflammation [144]. These lipids are produced by immune cells that partake in inflammation itself, as a failsafe to the deviant activation of pro-inflammatory cells and soluble agents (e.g., cytokines/chemokines, eicosanoids) that, if left unchecked, would lead to chronic inflammation and irreversible tissue damage [145] SPM biosynthesis—mostly achieved by monocyte/macrophages, neutrophils, platelets and hypoxic endothelia—occurs through the action of 5-, 12- and 15-LOX, but also that of acetylated COX2 and of CyP450, on the main polyunsaturated fatty acids of the plasm membrane, such as docosahexaenoic, and eicosapentaenoic acid (DHA, DPA and EPA, respectively), as well as AA [144]. This complex metabolic network gives rise to 5 main classes of SPM, namely DHA-derived D-series Resolvins (RvD), protectins and maresins (MaR), EPA-derived E-series resolvins (RvE) and AA-derived lipoxins (LX) [144,145]. During resolution, SPMs reduce neutrophil influx at the inflamed site and blunt the activity of pro-inflammatory phenotypes of monocyte/macrophages and lymphocytes, while promoting tolerogenic ones [90,144].

To our knowledge, a single work directly addressed the role of SPMs in the clinical phenotype of α-syn-overexpressing rat model of PD. PD1 treatment strongly reduced neuroinflammation and neurophysiological aberrancies in rats [146].

Given the potent immunomodulatory properties, SPMs represent a promising field in the research of therapeutic strategies for the treatment of synucleinopathies.

**Table 1:** Role of the main classes of endogenous bioactive lipids in synucleinopathies.

Lipid Class	Molecular Target	Effect	Reference
Eicosanoids	Prostaglandins	Increased production in animal models of ischemia after α-Syn ablation	[98]
	AA	Reduction in arachidonic acid incorporation and production in α-Syn knock out mice	[99]
	PGE_2_	Enhanced PGE_2_-induced toxicity in α-syn-overexpressing neurons.	[104]
		Enhanced α-Syn clear out in EP2^−/−^ microglia	[105]
		EP4 agonism hinders α-Syn oligomer-induced damage in primary microglia	[107]
	PGJ_2_	Accumulation of α-Syn in rodent brain and SK-N-SH cells	[109]
	5-LOX	Enhanced expression in DLB patients and mouse models	[112,113]
	CysLT1	Montelukast ameliorates DLB clinical phenotype and α-Syn brain load in DLB mouse models.	
		Clinical trial EudraCT: 2020-000148	[113]
Sphingolipids	GCase	It interacts with α-Syn and promotes its aggregation	[115,116,117]
		Mutations are associated with PD	
	S1P	Production and aggregation of α-Syn	[119]
	Ceramide		
	SphK1	SphK1 inhibition leads to neuronal death of dopaminergic neurons and to α-Syn overproduction	[120]
		Reduced activity in the MPTP-induced PD mouse model	[121]
	S1PR1	Fingolimod is beneficial in PD mouse models	[122]
	Acid Ceramidase	Inhibition leads to reduced α-Syn accumulation	[123]
		Polymorphisms of acid ceramidase genes (e.g., ASAH1) are associated with PD	[124]
	SMPD1	Control of sphingolipid metabolism and lysosomial burden that is linked with PD pathogenesis	[124,125]
	PSAP		
Endocannabinoids	CB_1_	Reduces bradikynesia in PD and enhances L-DOPA therapeutic effects	[127,128,129]
	CB_2_	Stimulation reduces loss of TH-positive neurons in pharmacological models of PD	[132,133]
		Upregulated in SNpc damage	[134]
	AEA, 2-AG	Elevated levels following SNpc damage	
	MAGL	Inhibition protects DA neurons in MPTP/probenecid-based PD models	[139]
	FAAH	Inhibition is protective in rodent models	[140,141,142,143]
SPMs	PD1	Neuroprotective in PD rat models	[146]

## 5. Targeting the Interaction of Alpha-Synuclein with Lipids for Discovering New Potential Biomarkers of Synucleinopathies

Increasing evidence strengthens the theory that PD and other synucleinopathies should not be categorized as single-entity diseases, but rather as part of a spectrum based on subtypes defined by underlying molecular mechanisms with corresponding signature biological features [147]. In this scenario, different pathogenetic pathways could be driven by the interaction of α-Syn with other proteins and with lipids. In the latter case, a key mechanism consists of an altered interaction of the protein with lipid membranes, which leads to α-Syn oligomerization [148] and to the subsequent formation of complex aggregates including fibrillar α-Syn and fragments of altered lipids membranes [40]. Understanding the interaction of α-Syn oligomers with lipid membranes can strongly have an impact on deciphering the molecular pathogenesis of synucleinopathies and might provide interesting insights for early diagnosis and disease-modifying therapies, by providing new possible strategies for discovering novel biomarkers based on a combined approach between proteomics and lipidomics [149].

As described in the previous section, α-Syn oligomers can often be found in proximity to lipid membranes, and associated with them at the level of synaptic vesicles or at the level of the mitochondria where they co-localize with highly curved inner mitochondrial membranes rich in cardiolipin [62]. On the other hand, monomeric α-Syn is mainly localized near the endoplasmic reticulum, the Golgi apparatus and at synaptic terminals, often associated with lipid rafts containing cholesterol, sphingomyelin, and gangliosides [150,151]. Oligomers taken up from the extracellular surroundings are targeted to lysosomes where they are subject to degradation [152]. The remaining particles are then stored in intracellular deposits, so-called ‘aggresomes’ [152]. It was found that PUFAs, generally present in lipid bilayers, induce the formation of oligomers, which subsequently leads to the formation of Lewy-like inclusions in mesencephalic (MES) cell cultures [153,154]. These data suggest that the accessibility to the lipid core, thus the low-grade saturation of the membrane, is a relevant factor determining oligomer-mediated membrane disruption.

Cholesterol has been demonstrated to lower the tendency of α-Syn oligomers to perturb lipid membranes by increasing membrane saturation. On the other hand, recent evidence reported that statins (in particular simvastatin), ameliorate the propagation of α-Syn oligomers and that cellular models in which intracellular accumulation of cholesterol is induced are characterised by an increased secretion of α-Syn aggregates [155]. Also, the lipoproteins content in the CSF has demonstrated an inhibitory effect on the seeding-competence of α-Syn oligomers, with low-density lipoproteins (LDL) (which are enriched in cholesterol) exerting a more powerful effect on α-Syn aggregation in respect to to high-density lipoproteins (HDL) [156]. It might be speculated that the intracellular accumulation of cholesterol could sequester oligomers within the cytoplasm, where they might exert neurotoxicity or drive their extracellular release in association with lipids droplets; conversely, extracellular lipoproteins might decrease the cell-to-cell transmission of α-Syn aggregates by preventing their access to the intracellular milieu. Previous studies have reported the close interaction of α-Syn with lipoproteins in plasma [157] and in CSF [158] and have highlighted how genetic polymorphisms in apolipoprotein composition correlate with the development of PD [159,160,161]. However, clinical studies have not identified any clear correlation between plasma levels of lipoproteins and the risk of developing PD [162], although in a recent study conducted on a cohort of 600,000 patients (AMORIS cohort), a potential protective effect of high plasma levels of triglycerides and LDL on the risk of developing PD has been reported [163]. Further analyses will be necessary in the future to reconcile this conflicting evidence to understand whether the binding of monomeric or oligomeric α-Syn to cholesterol and lipoproteins is beneficial or not in the progression and spreading of synucleinopathies.

Sphingolipids are preferentially bound by α-Syn at the plasma membrane [71,148,164,165,166,167]. As reported above in detail, altered sphingolipid metabolism, due to Glucocerebrosidase (GBA) gene polymorphisms increases the risk of synucleinopathy [116,168]. GBA is a lysosomal hydrolase that converts glucosylceramide or glucosylsphingosine into glucose and ceramide or sphingosine, respectively. A deficiency in GBA and the accumulation of glucosylceramide exacerbates α-Syn pathology promoting the formation of high molecular weight α-Syn species [169]. Besides genetic forms related to GBA polymorphisms, retromers (protein complex used in the recycling of transmembrane endosomal receptors to the *trans*-Golgi network) could be altered in some forms of idiopatic PD (iPD) and in the absence of a functional retromer network, sphingolipids in excess are diverted to lysosomes, leading to a dramatic increase in the production of ceramides [170]. The alteration in the lipid membranes due to the excess of ceramides further disrupts the retromers’ network, exacerbating neurodegeneration through a vicious circle [170], characterised by the further impairment of retromer activity [170] and the accumulation of ceramides in lysosomes and mitochondria. Heterozygous mutations in the retromer protein Vacuolar Protein Sorting 35 (VPS35) increase the risk of PD towards endo-lysosomal dysfunction and mitochondrial degeneration [171], with the accumulation of ceramides in degenerating neurons. Accordantly, the induced overexpression of VPS35 showed an improvement in motor symptoms and life expectancy in *LRRK2* and *Parkin* genetic models of PD [172]. Recent studies pointing to the application of lipidomics for the identification of novel PD biomarkers have focused on sphingolipid metabolism. Serum lipidome has reported reduced levels of sphingomyelins and ceramides in a cohort of 50 iPD patients in comparison to 45 age- and sex-matched HS, without correlation of these specific lipids with disease progression [173]. Extracellular and intracellular levels of different sphingolipids (including glucosylceramide, lactosylceramide, galactosylsphingosine and glucosylsphingosine) were assessed in a large cohort across GBA-associated PD (GBA-PD), idiopathic PD and HS. In the plasma of GBA-PD, glucosylceramide levels were slightly higher in comparison to iPD and HS, suggesting a possible application of this metabolite as a biomarker for the differentiation of GBA-PD from iPD [174]. In the Parkinson Progression Markers Initiative (PPMI) biomarkers of altered sphingolipid metabolism were invariably higher in the CSF of PD patients with increased values in patients carrying GBA mutations in comparison to patients GBA wildtype [175,176]. Altered ceramide metabolism has been also found in CSF-derived EVs of patients affected by different synucleinopathies [177], and increased ceramide levels in CSF correlate with the development of cognitive decline in iPD [178,179].

Oxidation and metabolism of PUFAs have been studied in correlation with various synucleinopathies. Lipid aldehydes, most commonly acrolein and malondialdehyde (MDA), can come from the oxidation of unsaturated fatty acid and have been reported to be elevated in various brain regions across synucleinopathies [148]. Furthermore, the type of elevated unsaturated lipids depended on the pathology. Lipid aldehydes 4-hydroxy-2-nonenal (HNE) and 4-oxo-2-nonenal (ONE) are elevated in the substantia nigra of PD brains [180], while MDA is elevated in PD, MSA and in the amygdala of AD brain with Lewy pathology (ADLD), where it is also associated with lipofuscin deposits in the hippocampal CA4 region. Moreover, in the amygdala, the levels of unsaturated phosphatidylethanolamine (PE) are tightly related to those of soluble α-Syn, while those of unsaturated phosphatidylserine (PS) are associated with pathological changes in the curvature of the plasma membranes [148]. Interestingly, the high prevalence of lipid aldehydes in the amygdala and the Substantia Nigra of PD and ADLB correlate with the high presence PUFAs in the neuronal plasma membranes of these brain regions [181,182]. Due to the heterogeneous distribution of lipid aldehydes and PUFAs in different anatomical regions, future studies could emphasise the possible application of the peripheral levels of lipid aldehydes, PUFAs and unsaturated phospholipids as possible biomarkers for the differential diagnosis of synucleinopathies. In this regard, recent lipidomics analyses on peripheral biofluids have detected a correlation between PUFA metabolic alterations, aging and cognitive decline [183,184]. Moreover, a two-sample Mendelian randomization study on a cohort of 33,674 PD patients and 449,056 healthy subjects detected a relationship between PUFA-related genetic variants and the risk of developing PD, whereas another study reported a significant association between PUFAs such as AA and EPA and an increased risk of onset of the same neurodegenerative disease [185].

Similarly, studies conducted on yeast cells overexpressing human α-Syn, and replicated in both rodents and human primary cells associated altered levels of monounsaturated fatty acids (MUFAs) with α-Syn-related dysfunctions and identified Stetoryl-CoA desaturase (SCD) as a possible therapeutic target for PD [186]; accordingly, SCD inhibitors rescued α-Syn pathology in vitro [187] and in vivo [188,189], preventing α-Syn phosphorylation and reestablishing the physiologic binding of this isoform to lipid membranes. Taken together, this evidence suggests that SCD polymorphisms might foster an increased risk of synucleinopathy.

As previously stated, PUFAs can be a substrate of Cyp450 oxidases, which include lipoxygenase (LOX), cyclooxygenase (COX), and cytochrome P450 (CYP) [190,191,192]. This pathway yields epoxy-PUFAs (Ep-PUFAs) that seem to have neuroprotective, anti-hypertensive, and analgesic effects [193,194,195]. However, the beneficial properties of Ep-PUFAs appear to be diminished when Ep-PUFAs are converted to their corresponding 1,2-diols by soluble epoxy hydrolase (sEH) [196]. sEH is expressed in different brain regions and multiple cell types such as astrocytes, endothelial cells, oligodendrocytes, neural cells, and microglia [197,198]. Several studies have demonstrated a correlation between sEH expression and phosphorylation of α-Syn, highlighting the role of sEH and Ep-PUFAs in the pathogenesis of synucleinopathies [199,200,201]. Moreover, animal models of Parkinsonism, obtained by 1-methyl-4-phenyl-1,2,3,6-tetrahydropyridine (MPTP) administration and presenting with neurotoxicity and α-Syn aggregates in dopaminergic and spinal motor neurons [202,203,204,205], reported an invariable activation of the sHE. Accordantly, sEH ablation and/or pharmacological inhibition protect neurons against MPTP-induced neurotoxicity in the mouse striatum [206]. In particular, TPPU (N-[1-(1-oxopropyl)-4-piperidinyl]-N′-[4-(trifluoromethoxy) phenyl)-urea), a potent sEH inhibitor, was found to be effective as an antagonist of MPTP-related mitochondrial dysfunction and in preventing dopaminergic neuron apoptosis [207]. Moreover, inhibition of sEH can mitigate also microglia activation, thus reducing neuroinflammation [208,209]. In accordance, sEH increased in the striatum of patients with DLB, where it correlated with the ratio of phosphorylated α-Syn to total α-Syn and with the markers of neuroinflammation [210]. Products of sEH, such as the 1,2-diols of the epoxy-PUFAs, could be considered as further possible biomarker candidates for the diagnosis and the clinical stratification of synucleinopathies. Interestingly, sEH isoforms (e.g., sEH2) have been linked with reduced activity of SPM lipids [211,212], suggesting that this enzyme might be involved in synucleinopathies by blunting the endogenous production of certain pro-resolving mediators.

## 6. The Importance of α-Syn–Lipids Interactions along the Gut-to-Brain Axis

### 6.1. Role of Short Chain Fatty Acids

Short-chain fatty acids (SCFAs) are organic fatty acids produced at the level of the colon through the fermentation of dietary fiber by commensal intestinal bacteria and play a crucial role in preserving intestinal homeostasis, as well as they can interact with the central nervous system (CNS) through the vagus nerve or the blood circulation [213]. Once produced, SCFAs are absorbed by the colon mucosa via the monocarboxylate transporter (MCT) and sodium-coupled monocarboxylate transporter (SMCT) [214]. Here, they can be used in mitochondria β-oxidation and the citric acid cycle to furnish energy for epithelial cells. The greatest part of the unmetabolised SCFAs are transported to the portal vein circulation, where the liver metabolises most of the propionate and butyrate, while about 10% of SCFAs are excreted through faeces [215,216]. Through this pathway, just a small amount of acetate, propionate, and butyrate can reach the systemic circulation. From the blood circulation, they will reach the brain as a privileged site where, due to their high lipid affinity and the high expression of SCFAs transporters, they easily pass the blood-brain barrier (BBB), concentrating into the nervous tissue [217]. Inside neurons, SCFAs can inhibit histone deacetylase (HDA) activity and according to preclinical data, HDAC inhibitors have been proven to have neuroprotective effects on α-Syn related neurodegeneration. For example, in MPTP-induced Parkinsonism, HDAC inhibitors enhanced the expression of GDNF (glial cell-derived neurotrophic factor) and BDNF (brain-derived neurotrophic factor) in astrocytes [218,219]. Moreover, sodium butyrate (SB), a SCFA metabolite, has been demonstrated to inhibit class I and II HDACs but not HDAC6 on a rotenone-induced PD model in Drosophila with an improvement of rotenone-induced cell death and locomotor impairment [220,221]. It has also been reported that SB is able to decrease apoptosis and degeneration of dopaminergic neurons in an α-Syn transgenic fly model, enhancing α-Syn acetylation [222,223]. In fact, the SB-mediated improvement is correlated with elevated brain dopamine levels and HDAC inhibitors can be considered applicable in the treatment of neurodegenerative diseases where acetylation homeostasis is significantly destroyed, leading to a reduction in the histone acetylation level and a disrupted the HDAC/HAT (histone acetyltransferases) balance [224]. For that reason, HDAC inhibitors may: (i) increase histone acetylation and support the expression of genes involved in cell survival and neuroprotection [225]; (ii) provide neuroprotection by blocking the release of proinflammatory cytokines and chemokines from microglia [226]; and (iii) increase the secretion of neurotrophic factors, reducing the expression of inflammatory factors. In addition, SCFAs can bind to G protein-coupled receptors (GPCRs) on the cell membrane, including GPR41/free fatty acid receptor 3 (FFAR3), GPR43/FFAR2, GPR42, and GPR109, as well as aryl hydrocarbon receptor (AhR) [227,228]. Through this binding, SCFAs can inhibit downstream NF-κB and MAPK signalling to weaken inflammation and increase AMPK signalling while inhibiting the mTOR pathway to sustain autophagy as well as enhance Nfr2 signalling to decrease oxidative stress [229,230]. In particular, FFAR2 has a higher affinity for SCFAs with shorter chains, whereas FFAR3 has a higher affinity for SCFAs with longer chains, like butyrate. They are plentiful in immune cells, adipose tissue, intestine, and bone marrow [231]. FFAR3 has been found in sympathetic ganglia to control sympathetic nerve activity and in brain endothelial cells [232,233]. In vitro experiments suggest that SB can protect dopaminergic cells from Salsolinol-induced neurotoxicity through the activation of FFAR3 [234]. Furthermore, studies have demonstrated that SB can contribute to neuroprotection by acting on FFAR2 and FFAR3 at the level of peripheral tissues, such as in enteric endocrine cells, supporting the secretion of glucagon-like peptide-1 (GLP-1) and improving the motor symptoms, the aggregation of toxic α-Syn species and the dopaminergic neurodegeneration induced by MPTP [235]. Additionally, osteocalcin (OCN), an osteoblast-secreted protein, could modulate brain activities with a neuroprotective role in Parkinsonian mice by increasing the production of propionate, which works as an FFAR3 agonist, blocking the dopaminergic neuronal loss [236]. In the end, since a significant reduction in the synthesis of SCFAs has been found in the faeces of PD patients [237], it is very important to deepen the protective effect and relationship between SCFAs against PD. A direct interaction between α-Syn and SCFAs has not been proved. However, it is well established that alterations in the microbiome’s composition impact the aggregation of α-Syn in the gut and the spreading of α-Syn aggregates from the gut to the brain [238,239]. In particular, recent pre-clinical evidence has demonstrated that dysregulated gut microbiota, as occurs in PD, might facilitate α-Syn aggregation in the gut [240]. Similarly, α-Syn aggregation in the gut mucosa can influence the composition of the microbiome and consequently the production of SCFAs [241]. The complex interplay between α-Syn, products of the microbiome and inflammation may disclose new possible therapeutic targets and biomarker candidates for synucleinopathies.

### 6.2. Interaction of α-Syn with Cholesterol and Lipoproteins—The Emerging Role of Hepatic Lipids Peroxidation

Abnormalities in lipid biology and in the interactions between lipids and α-Syn strongly contribute to the pathogenesis of synucleinopathies. In fact, recent epidemiologic evidence linked alterations of lipid metabolism with the development of PD and with α-Syn toxicity [242]. The formation of Lewy bodies is characterised by fibrillar α-Syn assembled with altered lipid membranes [243]: in particular, the molecular mechanism behind this process seems to depend on the physical-chemical and structural features of the membranes involved in this interaction, with different lipid classes being able to either promote or prevent the toxicity of α-Syn oligomers [244]. The incorporation of cholesterol within the lipid membranes is crucial in determining the interaction with amyloid α-Syn fibrils [245]. Indeed, neuronal cell membranes are characterised by high amounts of cholesterol that partake in many processes that affect their structure, plasticity and function [246,247]. Cholesterol is quite abundant in liquid-ordered microdomains of cell membranes containing specific proteins that seem to be responsible for α-Syn aggregation and neurodegeneration [65]. Interestingly, both toxic and genetic models of PD [180,248,249,250,251] have disclosed alterations in the main classes of lipids metabolised or synthesised in the liver, including fatty acids, sterols and sphingolipids. This supports the view that alterations in the lipid metabolism or in the assemblage of lipoproteins in the liver would contribute to the development of synucleinopathies in the brain. In particular, dysfunctional levels of cholesterol and cholesteryl esters can affect lysosomal activity, possibly impairing the maturation of late endosomes and lysosomes [252,253], with increased accumulation of misfolded α-Syn. In accordance, changes in the systemic levels of cholesterol and of its oxidated derivates, such as oxysterol-24-hydroxysterol (24-OHC) and 27-OHC, are closely associated with PD [254,255]. Oxysterols produced in the liver and delivered in the systemic circulation can easily cross the blood-brain barrier and noteworthy, different brain areas display well-defined 27-OHC/24-OHC ratios, e.g., ~1:8 in the frontal cortex, ~1:10 in the basal ganglia and 1:5 in the occipital cortex [254], with altered levels of 27-OHC being associated with both hypercholesterolemia and PD. Indeed, increased levels of 27-OHC have been found in the cerebral cortex of patients with PD [256], as well as having been proposed to affect the expression of α-Syn [257]. 27-OHC has been proposed to engage liver X receptors (LXR), which binds to the LXR response element in the α-Syn promotor, thus inducing the overexpression of the protein [258,259]. Moreover, α-Syn has been reported to interact with both the lipid and protein components of apolipoproteins [157]. Interaction with fatty acids and cholesterol occurs by the lipophilic domains of α-Syn [54,260]. α-Syn interacts either directly or indirectly with apoA1, apoE, and apoJ proteins, which compose HDLs. ApoA1 is involved in the regeneration of neuronal cells after damage [261] and is requested for cholesterol delivery to the brain; its plasma levels are decreased in PD patients as compared to healthy subjects [262]. On the other hand, other HDL-related apolipoproteins such as ApoJ and ApoE are abundant in the brain, and both interact with low-density lipoprotein receptor-related protein (LRP), the polymorphisms of which have been reported in PD [263,264]. Taken together, these data provide support for an association between α-Syn and HDL, probably towards the association of α-Syn with the intermediate lipoprotein fractions between HDL and LDL. This interaction may have relevant implications for the transport of α-Syn in the blood and across the BBB. Noteworthy, this mechanism could contribute to the spreading of α-Syn from the gut to the brain, exploiting the passage into the liver and the assembling of α-Syn oligomers with lipoproteins [265]. Further studies are needed to depict the intertwined mechanisms involving lipoprotein formation and inflammation in the spreading of α-Syn from the gut to the brain. On the other hand, the primary accumulation of α-Syn in the liver was also recently reported in both genetic and toxic models of Parkinsonism [266]. Liver neuropeptides are involved either in the liver or in neurodegenerative conditions [267,268,269,270]. Moreover, strong similarities have been disclosed between α-Syn related neurodegeneration and hepatic endoplasmic reticulum (ER) storage disorders, in which the ER stress could trigger the accumulation of misfolded proteins and the production of peroxidized lipids [271]. The possible production of inflammatory cytokines in the liver in response to α-Syn accumulation and lipids peroxidation represents a challenging and intriguing topic to investigate, which could bridge α-Syn aggregation, neuroinflammation and altered lipids metabolism in a common pathway along the gut-liver-brain axis. Future studies will decipher the complex role of the hepatic lipid’s peroxidation and ER stress in synucleinopathies.

Recent studies have underscored the significance of the gut–brain axis in PD, with mounting evidence suggesting that α-Syn pathology may originate in the gut and propagate to the brain via the vagus nerve [272,273,274]. This novel understanding of PD pathogenesis has shifted the research paradigm, highlighting the need to explore beyond the central nervous system and consider the roles of peripheral factors, such as intestinal lipids, in the initiation and spread of α-Syn aggregates. The interaction of α-Syn with lipids in the gut environment, characterised by a complex milieu of dietary and microbial lipids, presents an additional layer of complexity. These lipids can significantly impact the structural dynamics of α-Syn, affecting—as we have described in our review—its folding, aggregation, and interaction with cell membranes. Moreover, alterations in the composition of gut lipids, either through diet or changes in the microbiota, may influence the initiation of α-Syn pathology and its subsequent propagation to the brain. In particular, the role of lipids in the cell-to-cell transmission of α-Syn aggregates along the gut-brain axis warrants in-depth exploration. Lipid rafts, specialized microdomains on cell membranes rich in cholesterol and glycosphingolipids, have been implicated in the process of α-Syn aggregation and its intercellular transfer [65]. The interaction of α-Syn with these lipid rafts could facilitate its uptake by cells and its subsequent transport through the nervous system, highlighting a potential mechanism for the spread of pathology from the gut to the brain.

**Figure 3:**
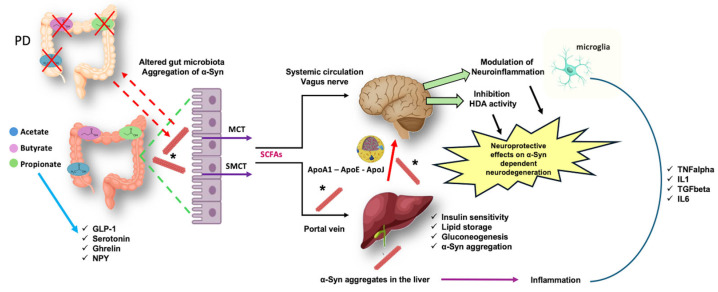
Schematic representation of pathways through which SCFAs may influence gut–brain axis. SCFAs are produced by the microbiota of the colon through the anaerobic fermentation of indigestible polysaccharides. Then, they are absorbed by epithelial cells, via H^+^-dependent monocarboxylate transporters (MCTs) or sodium-dependent monocarboxylate transporters (SMCTs). The greatest part of SCFAs is transported to the liver where they are metabolised. By the blood circulation or the vagus nerve, they can reach the brain inducing the secretion of gut hormones such as glucagon-like peptide 1 (GLP1) and serotonin (5-HT), NPY, or Ghrelin. SCFAs can cross the blood-brain barrier (BBB) and they may exert neuroprotective effects by inhibiting histone deacetylase (HDA) or modulating glial cells and neuroinflammation as well as the expression of α-Syn [228,280]. Alterations in gut microbiota, as occurs in PD, may induce α-Syn aggregates in the gut (*). Moreover, α-Syn aggregation in the gut epithelium can alter the composition of gut microbiota involved in the production of SCFAs [240]. In addition, α-Syn oligomers from the gut can reach the brain directly or exploit the passage into the liver, where they can form complexes with lipoproteins: ApoA1, ApoE and ApoJ [264]. Aggregates of α-Syn in the liver may also induce lipids peroxidation and liver inflammation with consequent production of pro-inflammatory cytokines affecting the brain.

On the other hand, liver plays a central role in synthesis, breakdown, and distribution of fatty acids and cholesterol. The interaction between α-Syn and lipids in the liver can influence lipid homeostasis, potentially leading to dysregulated lipid metabolism. This organ is highly susceptible to inflammation, a condition often exacerbated by an imbalance in lipid metabolism [275,276]. Conditions such as non-alcoholic fatty liver disease (NAFLD) and its more severe form, non-alcoholic steatohepatitis (NASH), are characterised by the accumulation of excess lipids in the liver, leading to inflammation and liver damage [277,278]. α-Syn can interact with lipid droplets and membranes in hepatocytes, influencing lipid accumulation and distribution [166,279]. These interactions may disrupt normal cellular functions, leading to cellular stress and the activation of inflammatory pathways. Moreover, α-Syn itself can undergo post-translational modifications, such as phosphorylation and oxidation, which may affect its binding affinity to lipids and its propensity to aggregate, as we have observed before. Aggregated or misfolded α-Syn can be recognized by the immune system as a danger signal, triggering inflammatory responses. This can lead to the activation of Kupffer cells, the resident macrophages of the liver, and the recruitment of additional inflammatory cells, exacerbating liver inflammation and damage. The connection between α-Syn, lipid metabolism, and inflammation in the liver also has implications for systemic inflammation and neuroinflammation. The liver plays a crucial role in regulating systemic immune responses; thus, liver inflammation can contribute to a pro-inflammatory state in the body. This systemic inflammation could, in turn, affect the brain, contributing to neuroinflammation and the progression of synucleinopathy. The gut–liver–brain axis provides, therefore, a potential pathway for the transmission not only of misfolded, pathological proteins but also of inflammatory signals from the gut to the brain and the importance of lipids in this circuit represents an intriguing and promising area of investigation. Figure 3 highlights the role of SCFA in α-Syn aggregation and the milestones of the gut-liver-brain axis in the propagation of α-Syn and inflammatory cytokines from the gut to the brain.

## 7. Conclusions

α-Syn aggregation is the pathological hallmark of PD and other synucleinopathies. Notwithstanding, therapies aimed at depopulation of α-Syn aggregates have not shown substantial efficacy in the treatment of motor and non-motor symptoms of these diseases, nor in improving their neuropathological and clinical progression. As a matter of fact, Lewy bodies contain a combination of filamentous α-Syn aggregates closely assembled with degenerating lipid membranes; the formation of Lewy bodies is an intricate molecular process in which the progressive degeneration of lipid compartments and the concomitant α-Syn aggregation contribute together to neuronal dysfunction. This evidence puts the α-Syn–lipid interactions at the crossroads between neuronal biology and neurodegeneration and calls for an integrated approach that goes beyond the biochemistry of α-Syn aggregation. The molecular structure of α-Syn is characterised by different lipid-binding domains and different conformers of both monomeric and aggregated α-Syn, disclosing a heterogenous potential in interacting with lipid membranes, modulating lipid metabolism and contributing to biological pathways mediated by bioactive lipids. Moreover, the interactions between α-Syn and lipoproteins at the level of the digestive tract might be determinant for the spreading of α-Syn pathological aggregates from the gut to the brain towards the bloodstream or the peripheral nerve fibers. Finally, the role of endogenous bioactive lipids that might modulate α-Syn aggregation by many mechanisms—including those that lead to the control of the neuroinflammatory surge—has been barely investigated to date and may provide in the near future important mechanistic and therapeutic information. All these mechanisms are currently poorly explored, curtailing the potential advances in providing new molecular biomarkers and disease-modifying therapies. Future studies should aim at elucidating the molecular mechanisms interconnecting different classes of bioactive lipids with α-Syn related pathology, also in light of different molecular and clinical phenotypes of patients affected by synucleinopathies, in which different and heterogenous interactions between α-Syn pathological species and lipids can occur. This approach would provide new strategies for the stratification of patients with synucleinopathies and the design of personalized therapies.

## Data Availability

Not applicable.
